# Case report: Individualized pulsed electromagnetic field therapy in a Long COVID patient using the Adaptive Force as biomarker

**DOI:** 10.3389/fmed.2022.879971

**Published:** 2023-01-11

**Authors:** Laura V. Schaefer, Frank N. Bittmann

**Affiliations:** ^1^Regulative Physiology and Prevention, Department of Sports and Health Sciences, University Potsdam, Potsdam, Germany; ^2^Practice of Integrative Medicine Bittmann, Potsdam, Germany

**Keywords:** individualized pulsed electromagnetic field, Adaptive Force, muscular holding capacity, case report, Long COVID, post-COVID syndrome, muscle weakness, fatigue

## Abstract

The increasing prevalence of Long COVID is an imminent public health disaster, and established approaches have not provided adequate diagnostics or treatments. Recently, anesthetic blockade of the stellate ganglion was reported to improve Long COVID symptoms in a small case series, purportedly by “rebooting” the autonomic nervous system. Here, we present a novel diagnostic approach based on the Adaptive Force (AF), and report sustained positive outcome for one severely affected Long COVID patient using individualized pulsed electromagnetic field (PEMF) at the area C7/T1. AF reflects the capacity of the neuromuscular system to adapt adequately to external forces in an isometric holding manner. In case, maximal isometric AF (AFiso_max_) is exceeded, the muscle merges into eccentric muscle action. Thereby, the force usually increases further until maximal AF (AFmax) is reached. In case adaptation is optimal, AFiso_max_ is ~99–100% of AFmax. This holding capacity (AFiso_max_) was found to be vulnerable to disruption by unpleasant stimulus and, hence, was regarded as functional parameter. AF was assessed by an objectified manual muscle test using a handheld device. Prior to treatment, AFiso_max_ was considerably lower than AFmax for hip flexors (62 *N* = ~28% AFmax) and elbow flexors (71 *N* = ~44% AFmax); i.e., maximal holding capacity was significantly reduced, indicating dysfunctional motor control. We tested PEMF at C7/T1, identified a frequency that improved neuromuscular function, and applied it for ~15 min. Immediately post-treatment, AFiso_max_ increased to ~210 *N* (~100% AFmax) at hip and 184 *N* (~100% AFmax) at elbow. Subjective Long COVID symptoms resolved the following day. At 4 weeks post-treatment, maximal holding capacity was still on a similarly high level as for immediately post-treatment (~100% AFmax) and patient was symptom-free. At 6 months the patient's Long COVID symptoms have not returned. This case report suggests (1) AF could be a promising diagnostic for post-infectious illness, (2) AF can be used to test effective treatments for post-infectious illness, and (3) individualized PEMF may resolve post-infectious symptoms.

## 1. Introduction

“Long COVID” receives increasing attention due to the high number of affected persons during SARS-CoV-2 pandemic. Six month post-infection 57% of COVID-19 survivors show one or more sequelae, after 1 year still half of them present at least one symptom (1, 2), regardless of infection severity (3). Long COVID shows similarities to myalgic encephalomyelitis/chronic fatigue syndrome (ME/CFS) (4–9), which is known since decades and can arise after viral infections (7–12). For post-infectious syndromes a dysfunction of the autonomous nervous system (ANS) was discussed to be the cause or at least a component (4, 7–9). The underlying mechanisms, the causality and the influence of pre-existing health conditions are not sufficiently known (1, 13). Innovative diagnostics and efficient causal therapies are urgently needed (14, 15).

Recently, Liu and Duricka reported sustained positive clinical outcomes for two Long COVID patients after stellate ganglion block (SGB), i.e., injecting local anesthetics near the stellate ganglion (4). Based on the rapid resolution of symptoms the authors concluded the “system needs to ‘reboot' to produce functional recovery” (4). The positive effect of SGB was suggested to be based on “sympathectomy,” which “produces its beneficial effects… by attenuating chronic sympathetic hyper responsiveness, improving cerebral and regional blood flow, and recalibrating the autonomic nervous system toward pre-COVID homeostasis” or “rebalancing the interaction between the nervous and immune system” (4).

Despite of delaying broad acceptance as valid treatment (4), therapeutic local anesthesia to sympathetic ganglia is supposed to be a promising approach for relieving severe conditions (16–20). It is applied since decades to treat several conditions, e.g., acute/chronic pain, functional disorders, dysautonomia, and chronic inflammation (16, 21). SGB, e.g., reduced the symptoms in patients with posttraumatic stress disorders (22, 23), may modulate the immune response (24), or stabilized ventricular rhythm (25). The local injection is claimed to be safe (4, 21), however, it is invasive and involves some risks (21, 26).

Another approach to influence the ANS is the use of weak, low-frequency pulsatile electromagnetic fields (PEMF) (27). Animal studies support the hypothesis that PEMF can be useful in therapy (27–30), e.g., in cardiac diseases (27, 30, 31). In humans, PEMF could normalize dysautonomia in children (32, 33) and was found to be effective to treat neuropathic/postsurgical pain and edema as well as several other indications (34–37). PEMF acupuncture of BL15 (bladder meridian and paravertebral T5) was found to activate the parasympathetic nervous system (38). Moreover, PEMF showed positive effects in cancer treatment (39). It modulated the physiology and electrochemistry of cancer cells and had immunomodulatory and systematic effects (39–41). PEMF was suggested to be a “suitable therapeutic approach with neuroimmunomodulatory, anti-inflammatory, anti-hyperglycemic, anti-hyperalgesic, and anti-allodynic actions” (35). Despite of those findings, development of PEMF therapy is slow due to the lack of scientifical evidence-based knowledge (36). Furthermore, the application parameters of PEMF were claimed to be “quite diverse, with no clear rationale for why particular parameters are chosen” (35).

Based on the above-mentioned knowledge and own clinical experience, we hypothesize (1) individualized PEMF in the sense of non-invasive neural therapy can be useful for treatment of dysautonomia in Long COVID; (2) the appropriate and helpful application parameters of PEMF can be tested by Adaptive Force (AF); (3) The AF can serve as biomarker (diagnostic/follow-up).

The AF characterizes the holding capacity of the neuromuscular system, which can be assessed, e.g., by a manual muscle test (MMT) objectified by a handheld device (42–44). During MMT, the tester applies a smoothly increasing force on the patient's limb in direction of muscle lengthening up to a considerably high force level. In case, the patient can adapt the muscle tension maintaining the isometric position during the entire force increase, the MMT is rated as “stable” and the maximal AF (AFmax) is reached under isometric conditions [AFmax = maximal isometric AF (AFiso_max_)]. An “unstable” adaptation is characterized by yielding of the limb during force increase. The patient is not able to adapt adequately. AFiso_max_ is considerably low and AFmax is reached during eccentric muscle action (43–45).

Healthy persons usually show stable adaptation ($\frac{AFiso_{\max}}{AFmax}\;\geq$ 99%) (43–45). Based on own practical experience, patients with, e.g., post-infectious syndromes show unstable adaptation. Common measurements of maximal strength (e.g., hand grip force) usually do not show a significant difference between patients and controls (46, 47). Two studies revealed a significantly reduced force in ME/CFS (48, 49). However, one did not describe sex effects. Females were overrepresented in ME/CFS group (96 vs. 62% in controls) (49), which might explain the lower strength. The findings are inconclusive and highlight that common maximal strength assessments might not be appropriate to investigate motor function in post-infectious states. We hypothesize AFiso_max_ might be a decisive motor function to investigate and uncover clear differences between patients and controls. Moreover, AFiso_max_ can react immediately to positive and negative inputs (43–45). A proposed neurophysiological explanation was given previously (42–45). Hence, the AF might be a useful biomarker to investigate patients and to determine helpful treatments, such as the individual PEMF.

This case report presents the positive clinical outcome for one Long COVID patient after a single treatment with individualized PEMF using the AF as biomarker.

## 2. Patient information

A 24-year-old female (168 cm, 65 kg; student since 2016; student assistant since 2020) presented herself in our practice of integrative medicine in August 2021. She reported a non-critical course of COVID-19 infection in December 2020 which lasted 2–3 weeks with symptoms as fever, loss of smell/taste, muscle pain and headache.

Afterwards she felt quite good for ~8 weeks. In March 2021 a state of Long COVID arose with severe symptoms as pronounced fatigue, fast exhaustion, post-exertional “crashs,” weakness, concentration problems, loss of speaking abilities, headache, muscle pain/cramps, sensitivity to stimuli (light/noise) and loss of smell. Less pronounced were nausea, nerve tingling, visual disturbances, memory, and sleeping problems and heavy perspiration. She was not able to proceed her Bachelor thesis, work as assistant or participate in social life. She appeared to be emotionally strong with good family bonding, although she naturally perceived her condition as very burdensome, especially because of the prospect of the clinicians she had to be patient, wait and pace herself.

She had a borreliosis infection in 2016. No other pre-existing health issues were reported (infections/hormonal/digestive/psychological). She always was sportive but sometimes not able to climb stairs in the current condition.

She already received exercise and physiotherapy, reflective breathing massage, tried supplements/vitamins and melatonin pills for sleeping problems. None of them led to a considerable condition improvement. Pacing herself resulted in a state in which she partly could resume work/studies. However, as soon as she went beyond her (low) limits (physically/cognitive/emotionally), a crash resulted (recovery: few days).

## 3. Clinical findings

**Figure 1:**
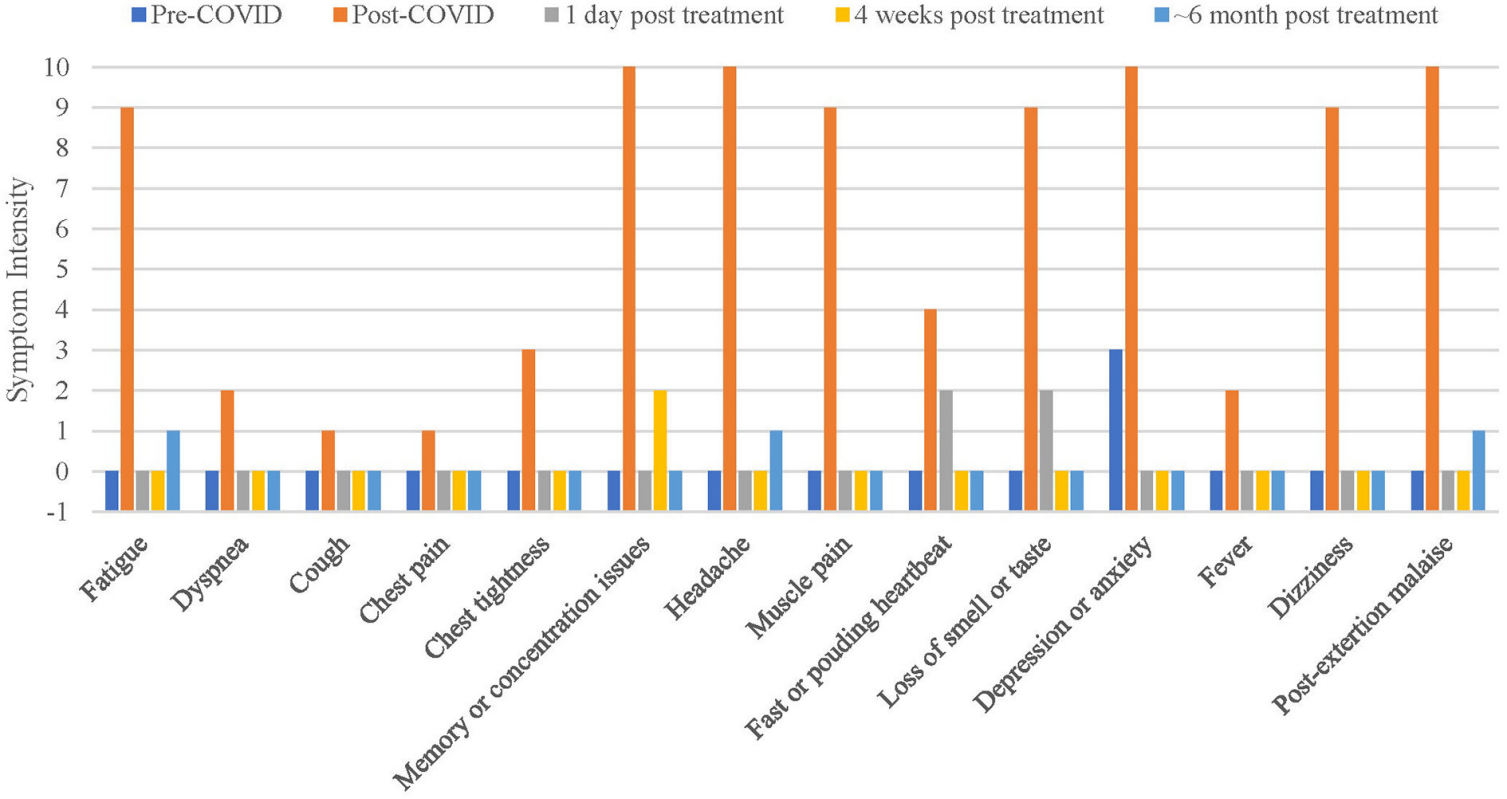
Intensity of Long COVID symptoms over time. Data was collected retrospectively for pre-COVID. Y-axis was set to −1 to visualize a score of zero [visualization was chosen following Liu and Duricka (4)].

The intensity of common Long COVID symptoms was inquired on a numerical scale [0-no to 10-very strong; according to Liu and Duricka (4)] retrospectively for pre-COVID baseline, during Long COVID (post-COVID) as well as 1-day, 4-weeks, and ~6-month post-treatment (Figure 1). Fatigue, memory/concentration issues, headache, muscle pain, loss of smell/taste, depression/anxiety, dizziness, and post-exertion malaise were rated by ≥ 9 post-COVID.

**Figure 2:**
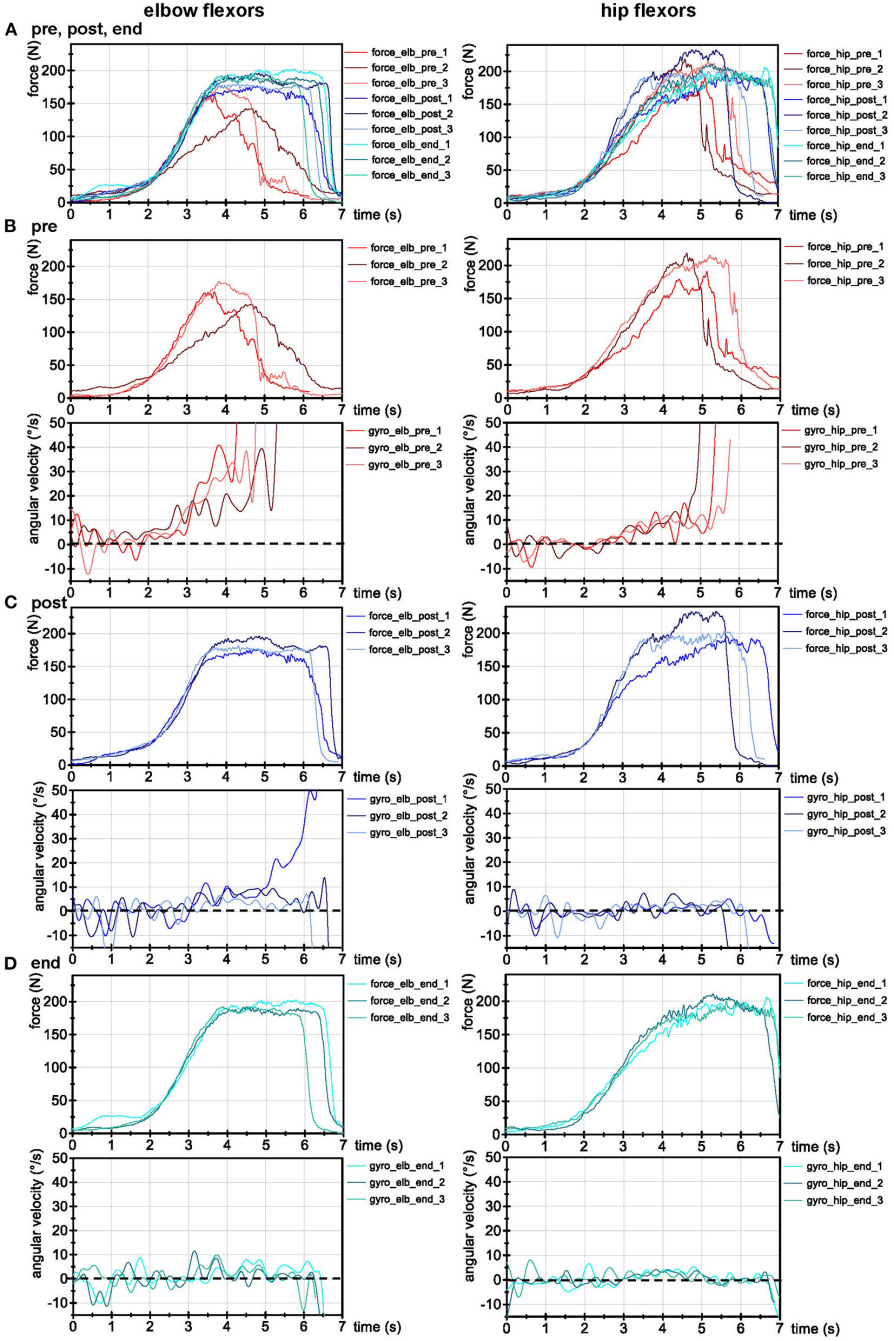
AF recordings of left elbow and hip flexors. **(A)** Force (N) of all trials before (pre), directly after (post) and 4 weeks after treatment (end). **(B)** Force (N) and angular velocity (°/s) of AF recordings pre-treatment, **(C)** directly post-treatment, and **(D)** 4-weeks post-treatment (end). All signals were filtered (butterworth; force: filter degree 5, cut-off frequency: 20 Hz; angular velocity: filter degree: 10, cut-off: 3 Hz). Dotted lines indicate zero for angular velocity.

For physical examination, the AF of nine different muscles/muscle groups was assessed on both sides by the MMT [hip flexors/adductors/abductors/extensors, foot dorsiflexors, pectoralis major (sternal and clavicular part), deltoid, and elbow flexors]. For left elbow/hip flexors, the AF was objectified (see below, Figure 2). All tested muscles showed a clearly unstable behavior in MMTs pre-treatment.

## 4. Timeline

Figure 3.

**Figure 3:**
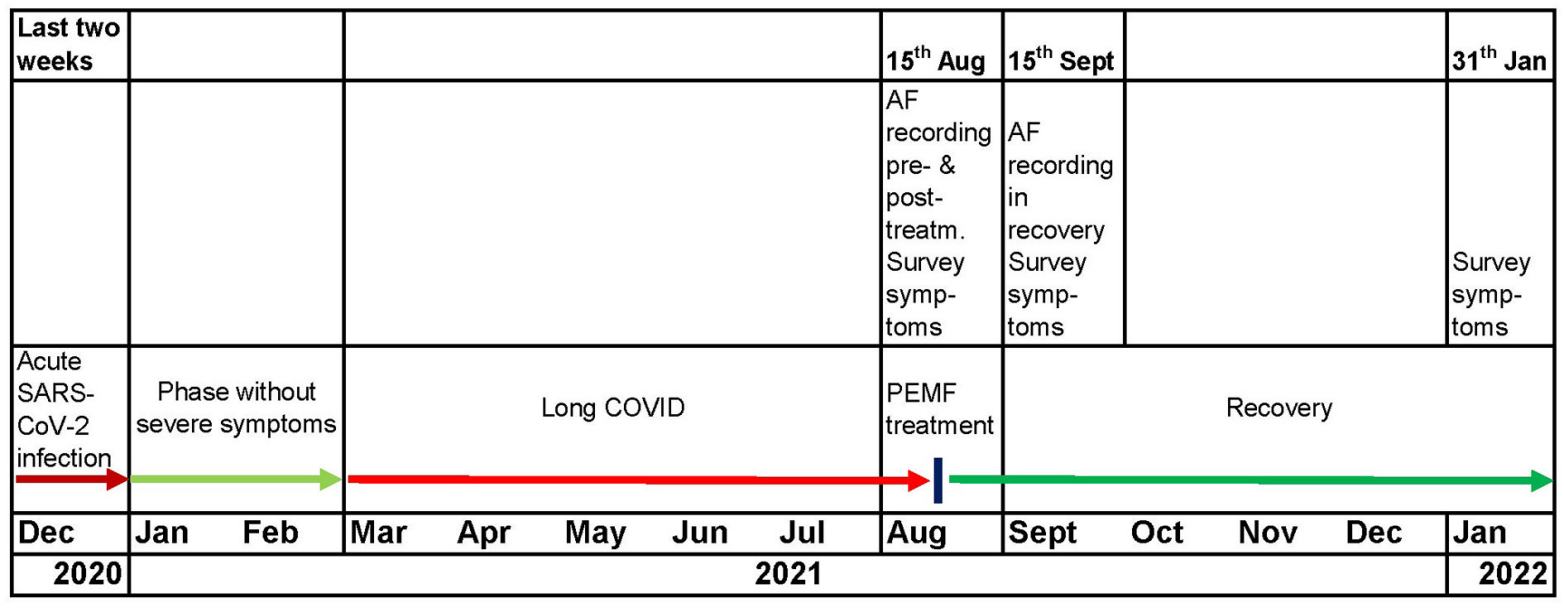
Timeline from acute SARS-CoV-infection over the ~6-month Long COVID period until the individualized PEMF treatment resulting into sustained recovery (~6-month post-treatment).

## 5. Diagnostic assessment

Diagnostic challenges for Long COVID appear because diagnosis is currently based on exclusion (15, 50). The patient provided documentation of a received extensive diagnostic assessment from a medical clinic (diagnosis: Long COVID). All other possible causes were excluded therein.

Besides the symptom intensity at five timepoints (Figure 1), the AF of left elbow and hip flexors was objectified by a handheld device which records reaction force (*N*) between tester and patient as well as limb position [angular velocity (°/s)]. It consists of strain gauges (co. Sourcing map, model: a14071900ux0076, precision: 1.0 ± 0.1%, sensitivity: 0.3 mV/V) and kinematic sensor technology (Bosch BNO055, 9-axis absolute orientation sensor, sensitivity: ± 1%) (42–45). Data were AD converted, buffered (sampling rate: 180 Hz) and sent (Bluetooth 5.0) to a tablet with measuring software (sticky notes). Data processing and evaluation were performed according to Schaefer et al. (43–45) in NI DIAdem 20.0 (National Instruments, Austin, TX, USA). Signals were interpolated (1 kHz) and filtered (Butterworth, filter degree 5, cut-off frequency 20 Hz). For visualization (Figure 2) the angular velocity was additionally filtered (degree: 3, cut-off: 10 Hz) to smoothen the oscillations (note: this leads to slightly different results between visual inspection in Figure 2 and results given below).

The following parameters were extracted: (1) AFmax (N): peak value of the whole trial. This can be reached either during isometric or eccentric muscle action. (2) AFiso_max_ (N): the maximal isometric AF refers to the highest force under isometric conditions. This was defined as the force at the moment in which the gyrometer signal increased above zero, indicating a yielding of the limb (breaking point). In case the gyrometer signal oscillated ~0 during the entire trial, AF_max_ = AFiso_max_. (3) Slope: the slope of force rise before AFiso_max_ of all trials was calculated by the difference quotient to control the increase. Reference points (time, force) were 70% and 100% of averaged AFiso_max_ of all as unstable assessed MMTs. The decadic logarithm was taken from values [lg(*N*/s)] since force rise is exponential. Arithmetic means (M) and standard deviations (SD) of each parameter were calculated of the three trials for each muscle and timepoint (Table 1).

**Table 1:** Results of Adaptive Force (AF) of left elbow and hip flexors.

	**AFiso**_**max**_ **(*****N*****)**			**AFmax (** ***N*** **)**			$\frac{AFiso_{\max}}{AFmax}$ **(%)**			**Slope [lg(** ***N*** **/s)]**		
	**Pre**	**Post**	**End**	**Pre**	**Post**	**End**	**Pre**	**Post**	**End**	**Pre**	**Post**	**End**
	**Elbow flexors**											
1	111.45	174.24	202.34	161.45	176.29	202.34	0.69	0.99	1.00	1.89	1.96	1.90
2	28.50	196.23	192.39	142.64	196.23	192.39	0.20	1.00	1.00	1.68	1.85	1.94
3	73.50	181.62	192.43	177.16	181.62	192.43	0.41	1.00	1.00	2.01	1.95	1.99
M	71.15	184.03	195.72	160.42	184.71	195.72	0.44	1.00	1.00	1.86	1.92	1.95
SD	41.52	11.19	5.73	17.28	10.33	5.73	0.25	0.01	0.00	0.17	0.06	0.05
CV	0.58	0.06	0.03	0.11	0.06	0.03	0.57	0.01	0.00	0.09	0.03	0.02
	**Hip flexors**											
1	-	194.70	206.05	190.83	194.70	206.05	-	1.00	1.00	1.59	1.98	1.91
2	71.33	232.72	211.09	218.49	232.72	211.09	0.33	1.00	1.00	1.66	1.87	1.86
3	51.76	202.60	199.68	215.53	202.60	199.68	0.24	1.00	1.00	1.81	2.08	1.69
M	61.54	210.01	205.61	208.28	210.01	205.61	0.28	1.00	1.00	1.69	1.98	1.82
SD	13.84	20.06	5.72	15.19	20.06	5.72	0.06	0.00	0.00	0.11	0.11	0.12
CV	0.22	0.10	0.03	0.07	0.10	0.03	0.22	0.00	0.00	0.07	0.06	0.06

Single values of each trial, the arithmetic means (M), standard deviations (SD), and coefficients of variation (CV) of the maximal isometric AF (AFiso_max_), the maximal AF (AFmax), their ratio (%), and of the slope of force rise [lg(N/s)] are given for each timepoint (pre: before treatment, post: directly after treatment, end: 4-weeks after treatment).

Figure 2 shows the signals of the three trials of left elbow/hip flexors at each timepoint (pre, post, and end), Table 1 shows the respective values. The entry MMTs were clearly unstable, indicated by low AFiso_max_ ≈ ~71 N (elbow) and ~62 N (hip). The muscle started to lengthen at ~44 ± 25% of AFmax (elbow) and ~28 ± 6% (hip). The slope was slightly smoother for pre vs. post vs. end (Table 1). Thus, the conditions for adaptation should have been even better in pre-tests.

After initial AF assessment, we tested the individual supportive PEMF frequency. For that, we placed the coil anteriorly centered to the area of stellate ganglion (C7/T1) and performed the MMT repeatedly whereby before each test we adjusted the frequency. As soon as the muscle showed stability, we used this frequency for treatment. The stabilized holding capacity indicates that exactly this configuration is supportive for the patient's system. Hence, the motor output leads us to the helpful PEMF frequency by instantaneously gaining stability. The PEMF has a reach of ~20 cm and, therefore, it had no special lateral effect.

Immediately after PEMF application (see below), all muscles were clearly stable in MMT. Results of AF values are given (Figure 2C, Table 1). The first trial of elbow flexors was not fully stable, indicated by a deviation of gyrometer signal above zero. However, the breaking point (AFiso_max_) was on a high force (174 N ≈ 99% of AFmax). All other trials post-treatment showed full stability with high AFmax reached during isometric conditions [M ± SD: $\frac{AFiso_{\max}}{AFmax}$ = 99.6 ± 0.7% (elbow); 100 ± 0% (hip)]. The isometric holding capacity was immediately increased by 2.6 (elbow) and 3.4-fold (hip) force compared to pre-treatment. The patient was able to maintain the isometric position of muscles during the entire force increase in contrast to pre-state. Those results support the manually assessed motor function as immediate reaction to the individual PEMF therapy.

## 6. Therapeutic intervention

Individualized PEMF therapy using bioMATRIX driver (Roland Pechan GmbH & Co.KG; sinusoidal signal, 100–1,000 Hz, max. 3 mT) was applied *via* coil to the area of C7/T1 assuming that it affects the stellate ganglion in order to “reboot” the ANS in the sense of a non-invasive neural therapy. The individual PEMF frequency of 550 Hz (flux density 1 mT) was tested by the AF and was applied for ~15 min. Established PEMF devices work with up to 10 mT (51). Only one treatment was performed since the condition improved immediately.

## 7. Follow-up and outcomes

The symptoms intensity improved immediately 1-day post-treatment and sustained until now (6-month post-treatment; Figure 1). The day after treatment she gave feedback (e-mail; translated): “I woke up this morning for the first time since months without a feeling of hangover. I don't have headache; my head feels broad and open (…). An incredible feeling. I don't have any nausea, I feel as 1,000 kg burden were removed from my body. I feel totally easy and energetic. I had no problems to fall asleep yesterday and slept through without melatonin pills. This morning I got out of bed without any difficulties, directly felt like doing Yoga and went for a bicycle trip.” She also felt like having “drunk 10 cups of coffee. I don't know where to go with my energy. It almost feels uncomfortable since my body is so twitchy.” It appears that the treatment led to sympathetic hyper activation. However, this adverse unanticipated reaction dissolved the next day.

Two weeks post-treatment she reported she still feels physically and mentally healthy. She was able to exercise as intensive as before COVID-infection (85 km bicycle trip without problems), she had no concentration issues and meetings with several persons were no problem anymore. “I am grateful and happy to have my life back.”

At follow-up appointment 4-weeks post-treatment, she felt well and healthy (Figure 1). All above-mentioned muscles showed stability in MMT, supported by AF recordings (Figure 2D, Table 1). The patient was able to stabilize the muscles in isometric holding conditions despite of the external increase until a considerably high AFiso_max_ = AFmax = 195.7 ± 5.7 N (elbow) and 205.6 ± 5.7 N (hip).

Approximately 6-weeks post-treatment she received a lymph drainage (head and shoulder girdle) independent of our intervention and reported of headache, fatigue, concentration problems and sensitivity to stimuli afterwards for 3 days. She had another appointment in our practice ~1 week later. The muscles were still stable in entry MMTs. They became unstable after lymph drainage performed in our practice indicating that it irritated her system. By applying an individually newly tested PEMF frequency (590 Hz) the muscles were stabilized again. After the next lymph drainage independent of our intervention, she perceived headache for 1 hour but felt well afterwards. Approximately 10-weeks post-treatment she reported “I feel currently wonderful”. The sustainability was underpinned by the last assessment (January 2022; Figure 1). She reported, she is physically completely on the level before COVID, “if not better.” However, after emotional stress fatigue sometimes returns, but not in the previous extent.

## 8. Discussion

This case report suggests that low-frequency PEMF to the area of stellate ganglion with individually tested frequency using the AF might be an effective therapy in Long COVID patients. Since Liu and Duricka found a similar outcome after SGB (4), we assume that PEMF to the area C7/T1 affect the stellate ganglion. Based on our case and their suggestion that “cervical sympathetic chain activity can be blocked with local anesthetic, allowing the regional autonomic nervous system to ‘reboot”' (4), we propose the same effect might be gained by individualized PEMF therapy. A rationale for the mechanisms behind the hypothesis rebooting the ANS was given by Liu and Duricka (4).

The benefit of PEMF is that it is non-invasive, the patient does not feel anything of the intervention (see below) and no side effects are known (36, 39, 52). However, a successful treatment will not be that easy in every Long COVID patient. Some will have more severe pre-existing health issues which might hinder the positive outcome of a single treatment. From our current experience, three main factors in Long COVID occur: dysautonomia, pre-existing, and/or current mental stress and previous infections affecting the lymphatic system, which might lead to lymphatic entrapments post-COVID. Based on psychoneuroimmunology it is known that those factors interact (53). This is underpinned by the present case, since the patient relapsed after lymph drainage but could be switched back by one re-treatment. The switching between both states as immediate responses to disturbing or helping interventions speaks for a regulative character of Long COVID condition, at least in part. This would explain the instant reversibility observed in some cases. It is suggested that the complex psychoneuroimmunological network might still be vulnerable after “rebooting” the ANS. Lymphatic and mental stress might impede an immediate positive outcome or lead to a relapse. Consequently, such conditions must be treated, too. However, the ANS dysfunction—presumably triggered by SARS-CoV-2 infection—could benefit from individualized PEMF therapy determined by the holding capacity (AFiso_max_) of the neuromuscular system.

AFiso_max_ was suggested to be especially sensitive regarding interfering inputs entering the complex motor control processes. At least the thalamus, cerebellum, inferior olivary nucleus, red nucleus, basal ganglia, cingulate cortex, and the sensorimotor cortex are involved in processing adaptive motor control (54–95). Due to the strong interconnections between those areas (73, 84) and since they also process other inputs (e.g., emotions/nociception) (63, 65, 68, 69, 73, 96–99), it was proposed that the motor output in the sense of AFiso_max_ can be modified by different stimuli—positive and negative ones. The pro-inflammatory cytokine/chemokine profile (100), organ damage, lymphatic stress and/or the dysautonomia in Long COVID might impair that motor function. In case this is based on malfunction, it can be resolved immediately by applying the helpful therapy, e.g., individualized PEMF. The instantaneous improvement of AFiso_max_ by 2.6 and 3.4-fold by applying the individualized PEMF frequency clearly demonstrated this. This effect cannot be reached by training. It must be the result of a functional readjustment of the patient's system. In contrast to maximal forces (as AFmax or MVIC), which can be reached also in dysfunctional state [as found here or in other studies (46, 47)], the holding capacity might uncover the dysfunction. In case the patient must adapt in an isometric holding manner to an increasing external force, the maximal force cannot be demanded under isometric conditions anymore. The adjustment of tension under stable muscle length fails and the limb gives way on significantly low forces. The AFiso_max_ improved though immediately by applying the helpful PEMF. As was postulated by Mert (35), there is no rationale which PEMF parameters should be applied. So, why not “ask” the patient's system? The holding capacity seems to lead the way to the individual helpful parameters. Applying any frequency would not have this positive effect. Therefore, it is necessary to test the PEMF frequency individually by adequate biomarkers, as the neuromuscular holding capacity.

## 9. Conclusion

In conclusion, we suggest (1) to include pre-existing health issues of Long COVID individuals, especially concerning mental stress and previous infections and to examine the lymphatic system regarding flow restrictions. (2) The AF provides a valuable biomarker which can be used as functional diagnostic parameter for patients in post-infectious states, to determine the individual appropriate cause-related therapy and to monitor follow-up, since it seems to correlate with the patient's condition. (3) Soft, low-frequency PEMF with an individually tested frequency for each patient at the actual timepoint seems to be useful to “reboot” the dysfunctional ANS and might be an alternative non-invasive neural therapy. Further research is needed to verify and pursue this approach.

## Data availability statement

The original contributions presented in the study are included in the article/supplementary material, further inquiries can be directed to the corresponding author.

## Ethics statement

Ethical review and approval was not required for the study on human participants in accordance with the local legislation and institutional requirements. The patients/participants provided their written informed consent to participate in this study. The patient gave written informed consent to publish her case.

## Author contributions

FB performed the measurements. LS analyzed the data and wrote the manuscript. All authors planned and designed the treatment process and measurements of the patient. All authors critically revised the manuscript and approved it for publication.
